# Shedding Light on Avian Influenza H4N6 Infection in Mallards: Modes of Transmission and Implications for Surveillance

**DOI:** 10.1371/journal.pone.0012851

**Published:** 2010-09-20

**Authors:** Kaci K. VanDalen, Alan B. Franklin, Nicole L. Mooers, Heather J. Sullivan, Susan A. Shriner

**Affiliations:** National Wildlife Research Center, Wildlife Services, Animal and Plant Health Inspection Service, United States Department of Agriculture, Fort Collins, Colorado, United States of America; Veterinary Laboratories Agency, United Kingdom

## Abstract

**Background:**

Wild mallards (*Anas platyrhychos*) are considered one of the primary reservoir species for avian influenza viruses (AIV). Because AIV circulating in wild birds pose an indirect threat to agriculture and human health, understanding the ecology of AIV and developing risk assessments and surveillance systems for prevention of disease is critical.

**Methodology/Principal Findings:**

In this study, mallards were experimentally infected with an H4N6 subtype of AIV by oral inoculation or contact with an H4N6 contaminated water source. Cloacal swabs, oropharyngeal swabs, fecal samples, and water samples were collected daily and tested by real-time RT-PCR (RRT-PCR) for estimation of viral shedding. Fecal samples had significantly higher virus concentrations than oropharyngeal or cloacal swabs and 6 month old ducks shed significantly more viral RNA than 3 month old ducks regardless of sample type. Use of a water source contaminated by AIV infected mallards, was sufficient to transmit virus to naïve mallards, which shed AIV at higher or similar levels as orally-inoculated ducks.

**Conclusions:**

Bodies of water could serve as a transmission pathway for AIV in waterfowl. For AIV surveillance purposes, water samples and fecal samples appear to be excellent alternatives or additions to cloacal and oropharyngeal swabbing. Furthermore, duck age (even within hatch-year birds) may be important when interpreting viral shedding results from experimental infections or surveillance. Differential shedding among hatch-year mallards could affect prevalence estimates, modeling of AIV spread, and subsequent risk assessments.

## Introduction

Avian influenza viruses (AIV) is a term used to describe influenza Type A viruses, which have been isolated from a wide range of avian species throughout the world [1]. Wild birds, predominantly waterfowl and shorebirds, serve as the natural reservoir of influenza A viruses [2]. While AIV rarely cause disease in their wild bird hosts, the potential transmission of AIV to hosts of agricultural and human health importance is of concern. While some AIV can be directly transmitted from wild birds to domestic birds and/or mammals [3], [4], [5], AIV strains may also recombine with mammalian-derived influenza strains, producing recombinant influenza viruses capable of causing disease in humans, and other species [4], [5], [6].

Surveillance of AIV in wild birds may be used to produce risk assessments for poultry, humans, swine, and other animals. AIV generally replicates in the respiratory and/or digestive tracts of infected wild birds suggesting that infections may be monitored with oropharyngeal swabs, tracheal swabs, cloacal swabs, or environmental samples such as water and feces [7], [8], [9]. However, when conducting a large-scale surveillance effort, the collection of so many different sample types can be arduous and time consuming for field personnel. Similarly, AIV isolation in the laboratory can be lengthy, expensive, and AIV strain dependent. Recently, real-time reverse-transcription polymerase chain reaction (RRT-PCR) has been evaluated for rapid detection of AIV [10], [11], [12], [13], [14], [15]. While RRT-PCR cannot determine the infectiousness of samples, the molecular assay is quicker, more sensitive and less expensive than virus isolation for AIV screening [14]. A major objective of this study was to determine the best sampling method available for AIV detection by RRT-PCR. The AIV subtype H4N6 is one of the most common subtypes found through surveillance of wild waterfowl in North America [1], [16], [17] and a wild bird isolate of an H4N6 virus was used in this study to infect mallards (*Anas platyrhychos*). Various samples (oropharyngeal swabs, cloacal swabs, fecal samples, and water samples) were collected and evaluated for the ability to detect virus.

Additionally, because AIV is often shed in feces, and it has been shown to survive for extended periods of time in laboratory water [7], [8], [18], [19], it has been hypothesized that water is one medium through which wild birds both transmit and acquire the virus [7], [8], [18], [19], [20], [21]. An additional objective of this study was to investigate shared water sources as potential routes of AIV H4N6 infection of mallards.

## Materials and Methods

### Ethics Statement

All experiments were approved by the Institutional Animal Care and Use Committee of the United States Department of Agriculture, Animal and Plant Health Inspection Service, Wildlife Services, National Wildlife Research Center (NWRC), Fort Collins, CO, USA. (Approval number NWRC 1477).

### Study Species

Mallards (ages 3 mo and 6 mo) were purchased from two game bird farms (Game Birds Unlimited, Longmont, CO, USA and Field Trial Game Birds, Fort Collins, CO, USA). Ducks were quarantined for two weeks and the NWRC animal care husbandry guidelines for waterfowl (SOP AC/CO 028.00) were followed. All mallards were negative for antibodies to influenza A viruses by an epitope-blocking ELISA[22]. Throughout the experiment ducks were housed in an indoor aviary as cohorts of three or four ducks per pen. Each pen contained a shallow water bowl, food bowl, and a 375 L oval stock tank (Rubbermaid, Atlanta, GA) filled with water for swimming and preening.

### Oral inoculation and sample collection

Fifteen mallards were each orally inoculated with 1 mL of approximately 10^6^ EID_50_ of AIV (A/wildbird/PA/185996-06/07(H4N6)). The strain was passaged only once through chicken embryos to limit genetic mutations that may cause it to become less adapted to wild birds. Cloacal swabs and oropharyngeal swabs were collected from each duck daily through 8 days post inoculation (dpi). Six fresh fecal samples were collected daily from the floor of each pen and a 50 mL water sample was collected daily from the water column of each water tank through 9 dpi. Fecal samples were originally collected on a spatula and weighed. However to facilitate comparisons with oropharyngeal and cloacal swabs, we determined that an average fecal swab contained 0.0929±0.0089 g of feces. We present our fecal sampling results as ‘fecal swab equivalents.’

### Inoculation via shared water source and sample collection

On 5 dpi, six orally inoculated ducks were relocated from their two original pens to two new pens and sampling continued as stated above (see *Oral inoculation and sample collection* section). All surfaces in the two original pens were decontaminated with a 10% bleach solution leaving only the water in the water tanks as a potential source of virus in the pen; eight naïve ducks were introduced the following day. Cloacal swabs, oropharyngeal swabs, eight fecal samples, and a 50 mL water sample were collected daily for an additional 6 days where 0 dpi was the day of introduction to the contaminated water source.

### Virus Detection

All samples were initially tested by RRT-PCR for viral RNA detection and quantification. RNA was extracted using the MagMAX-96 AI/ND Viral RNA Isolation Kit (Ambion, Austin, TX). Primers and probe specific for the influenza type A matrix gene developed by Spackman, et al [10] were used in conjunction with the ABI one-step RT-PCR master mix and the ABI 7900 Real Time PCR system (Life Technologies Corp, Carlsbad, CA) with thermocycler conditions developed by Agüero et al [23]. Calibrated controls with known viral titers (10^2^ EID_50_/mL–10^5^ EID_50_/mL) were also analyzed with RRT-PCR to construct 4-point standard curves. Sample viral RNA quantities were extrapolated from the standard curves and presented as PCR EID_50_ equivalents/mL. Virus isolations (VI) in MDCK cells and/or chicken embryos were performed following published protocols [24] on a subset of water samples and cloacal samples. Isolations were conducted to confirm the presence of infectious virions in a subset of samples; viral titers were not calculated due to the cost of assays and low concentrations.

### Statistical Analysis

All statistical analyses were conducted using SAS® (Version 9.1 for Windows, SAS Institute, Inc., Cary NC, USA). Statistical methods were developed to estimate the effect of sample type (oropharyngeal swab, cloacal swab, or fecal sample), route of infection (oral inoculation or water transmission) and duck age (3 mo or 6 mo) on viral RNA shedding rates. Specifically, viral RNA shedding over the course of infection was analyzed using multiple linear regression based on maximum likelihood estimation. Analyses were performed using PROC GENMOD in SAS, specifying a normal distribution and a log link. Because we did not have individual duck identification for fecal samples, all data were analyzed by pen. The mean sum of viral RNA shed over eight days across all ducks in each pen was used as the dependent variable. Pens contained a variable number of 6 mo and 3 mo ducks, so we used the percent of 6 mo ducks in the pen as a proxy for testing the impact of duck age on virus concentrations. In addition to the three main explanatory variables (sample type, route of infection, age) all two- and three-way interactions between the main variables were also included in the statistical models.

Multiple logistic regression (PROC LOGISTIC) was used to estimate the probability of AIV detection for cloacal swabs, oropharyngeal swabs, and fecal swab equivalents over the course of infection. The purpose of this analysis was to compare the efficacy of the different swab types for AIV detection. Therefore, we used the positive/negative result for each swab rather than the sample concentrations as above in order to determine the probability of detecting a positive sample for each sample type. The models tested four variables: 1. sample type (oropharyngeal, cloacal, fecal), 2. dpi, 3. percent of 6 mo ducks in a pen, and 4. route of infection (experimental inoculation, water transmission) as well as all two way interactions and a quadratic term for dpi to capture the polynomial shape of the shedding curve over time.

In both analyses, information-theoretic model selection was used to identify the model that best explained the information in the data [25]. Models were ranked using a second order bias correction version of Akaike's Information Criteria for small sample sizes (AICc), where minimum AICc indicated the best model explaining the data. We used Akaike weights as a measure of the likelihood for each model, given the data, relative to other models examined [25].

## Results

### Viral RNA shedding in orally inoculated ducks

**Figure 1:**
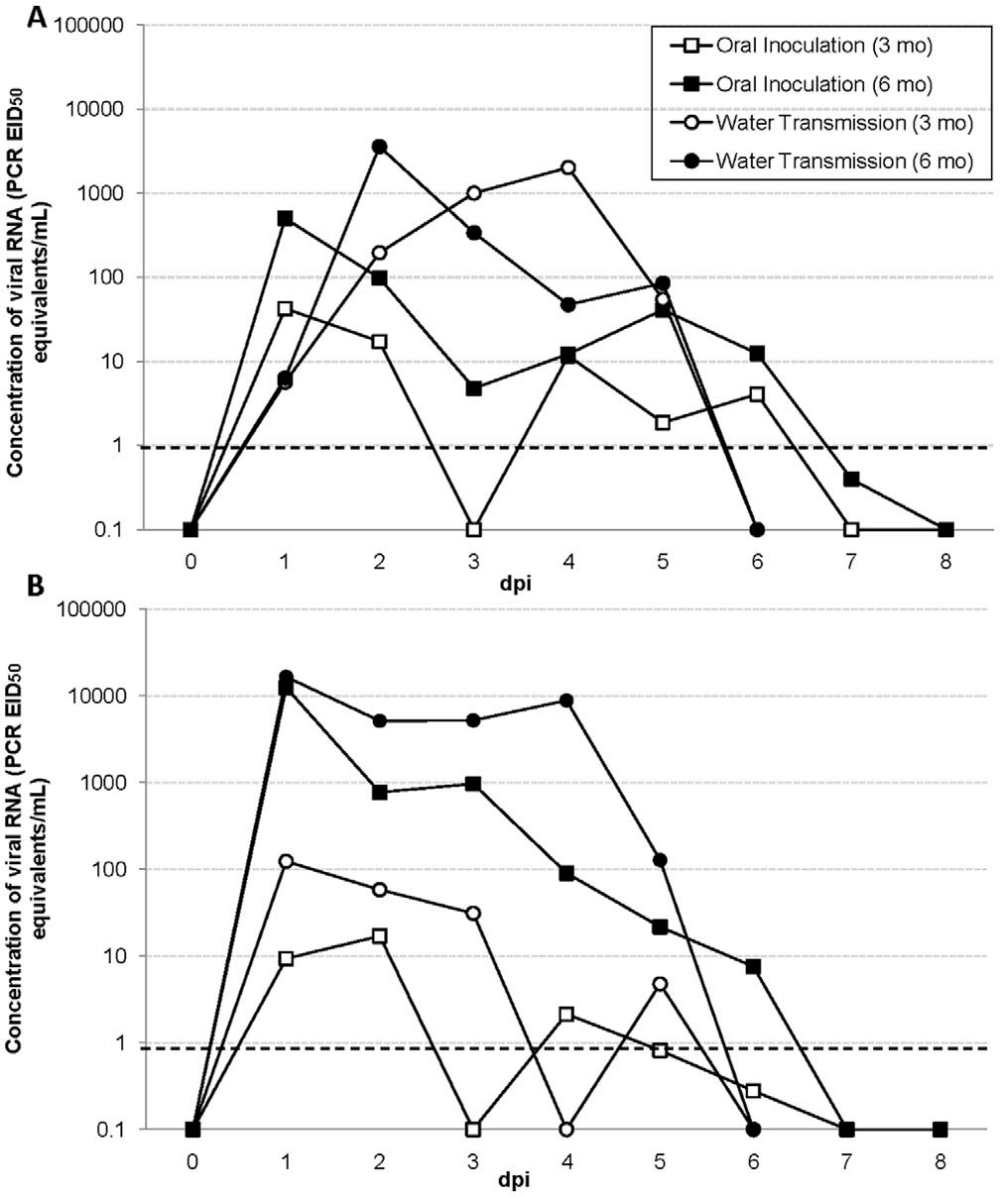
Mean shedding of H4N6 AIV RNA by mallards infected via oral inoculation or water transmission. (**A**) Cloacal shedding, (**B**) Oropharyngeal shedding. RRT-PCR was used to detect and quantify viral RNA in cloacal and oropharyngeal swabs. Values were extrapolated from a standard curve and presented as PCR EID_50_ equivalents/mL on a log scale. Each point represents the arithmetic mean of 6 mo mallards (n = 11) or 3 mo mallards (n = 12). On average, the shedding curves differed between 6 mo and 3 mo mallards. The dashed line represents our RRT-PCR threshold of detection as 10^0^ PCR EID_50_ equivalents/mL.

The threshold for detection of our RRT-PCR assay was determined to be approximately 10^0^ EID_50_. During validation, it was at this concentration where at least 50% of replicates were positive (data not shown). Viral RNA was detected in cloacal swab samples from 13/15 ducks for an average of 2.0 days (SE = 0.3) in 3 mo ducks and 4.1 days (SE = 0.3) in 6 mo ducks (Figure 1A). On average, viral RNA concentrations in positive cloacal swabs peaked at 10^1.3^ PCR EID_50_ equivalents/mL (Range of individual peaks = 10^0.5^–10^2.0^) in 3 mo ducks and 10^4.1^ PCR EID_50_ equivalents/mL (Range of individual peaks = 10^2.2^–10^4.9^) in 6 mo ducks. Viral RNA was detected in oropharyngeal swabs in all orally inoculated ducks for an average of 2.3 days (SE = 0.5) in 3 mo ducks and 4.1 days (SE = 0.5) in 6 mo ducks (Figure 1B). On average, the viral RNA concentrations detected in oropharyngeal swabs peaked at 10^1.6^ PCR EID_50_ equivalents/mL (Range of individual peaks = 10^0.4^–10^2.3^) in 3 mo ducks and 10^2.7^ PCR EID_50_ equivalents/mL (Range of individual peaks = 10^1.5^–10^3.3^) in 6 mo ducks. Viral RNA was also detected in at least one fecal sample from each pen for an average of 6.8 days (SE = 0.6) with a mean peak viral RNA concentration among pens of 10^3.5^ PCR EID_50_ equivalents/mL (Range of individual pen peaks = 10^1.4^–10^4.3^) on approximately 3.0 dpi (SE = 0.3).

### Transmission of H4N6 via a shared water source

**Figure 2:**
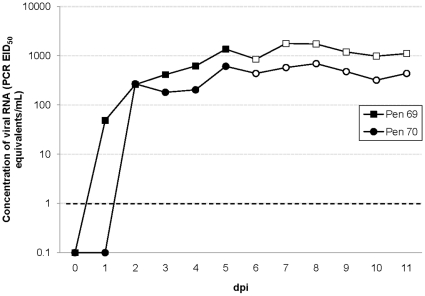
AIV RNA detected in water tanks. AIV RNA was detected and quantified by RRT-PCR in the water tanks housing orally-inoculated mallards (represented by solid markers). Quantities were extrapolated from a standard curve and presented as PCR EID_50_ equivalents/mL on a log scale. On 5 dpi, mallards were removed from two of the pens (69 and 70). These pens were disinfected, leaving only the AIV contaminated water in water tanks. When naive ducks were exposed to the AIV contaminated water, they became infected and maintained the virus concentration (represented by open markers) for the duration of the experiment. The dashed line represents our RRT-PCR threshold of detection as 10^0^ PCR EID_50_ equivalents/mL.

Ducks were removed from two of the pens which had viral RNA quantities of 10^3.1^ PCR EID_50_ equivalents/mL and 10^2.8^ PCR EID_50_ equivalents/mL in their water tanks. Four naïve ducks were introduced to each pen and viral RNA was detected from cloacal and oropharyngeal swabs of all eight ducks after introduction to the contaminated water. Viral RNA was detected in cloacal swabs for an average of 2.3 days (SE = 0.7) in 3 mo ducks and 4.3 days (SE = 0.3) in 6 mo ducks (Figure 1A). The virus concentrations detected in cloacal swabs from 3 mo ducks peaked at approximately 10^2.1^ PCR EID_50_ equivalents/mL (Range of individual peaks = 10^1.3^–10^2.7^) while the virus concentrations in 6 mo ducks peaked at approximately 10^4.2^ PCR EID_50_ equivalents/mL (Range of individual peaks = 10^3.6^–10^4.6^). Viral RNA was detected in oropharyngeal swabs for an average of 3.8 days (SE = 0.3) in 3 mo ducks and 4.7 days (SE = 0.2) in 6 mo ducks (Figure 1B) with viral RNA concentrations that peaked at approximately 10^3.3^ PCR EID_50_ equivalents/mL (Range of individual peaks = 10^1.6^–10^3.8^) and 10^3.6^ PCR EID_50_ equivalents/mL (Range of individual peaks = 10^1.8^–10^4.0^) respectively. Viral RNA was also detected daily in at least three fecal samples per pen with a mean peak viral RNA concentration among pens of 10^4.4^ PCR EID_50_ equivalents/mL (Range of peak shedding = 10^4.2^–10^4.5^) for the duration of the study. Viral RNA concentrations detected in the water tanks were also maintained (Figure 2). No viral RNA was detected in any samples (swabs, feces, or water) for two negative control ducks co-housed in a nearby pen.

### Virus isolation

In order to further demonstrate transmission of AIV from orally inoculated ducks through water to naïve ducks, we documented the presence of infectious virions in swab and water samples. Although several studies have shown that AIV isolation is unsuccessful in confirming RRT-PCR positive results approximately 25–35% of the time [14], [23], [26], [27], we were able to confirm the presence of infectious virus throughout the suspected transmission cycle. Due to the cost and time associated with AIV isolation in chicken embryos, we initially tested all samples by virus isolation in MDCK cells. At least one cloacal swab from each of the six orally-inoculated ducks tested positive for infectious AIV in MDCK cells. Similarly, at least one cloacal swab from 6/8 introduced ducks tested positive for infectious AIV after they were exposed to the shared water source. Water samples collected from the two water tanks were negative for infectious AIV in MDCK cells but positive for infectious AIV after two passages in chicken embryos.

### Comparison of Sampling Methods

**Figure 3:**
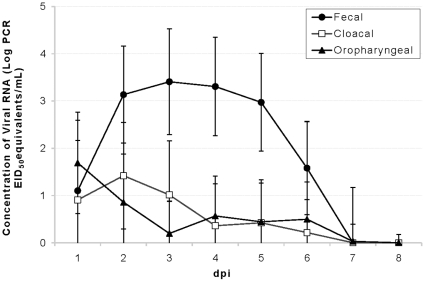
Mean H4N6 AIV RNA detected using 3 different sampling methods. AIV RNA quantities detected in cloacal swabs, oropharyngeal swabs, and fecal samples were compared. Because fecal samples were originally weighed (not swabs), we determined that an average fecal swab contained 0.0929±0.0089 g of feces and present these data as ‘fecal swab equivalents.’ Estimated virus concentrations in fecal swab equivalents were higher than the concentrations detected in cloacal and oropharyngeal swabs regardless of the age of ducks or route of infection. Error bars represent standard errors.

Based on minimum AICc, the best multiple linear regression model identified for the mean sum of viral RNA shed through 8 dpi included sample type, duck age, and route of infection and an interaction between duck age and route of infection. This result indicates that sample type, duck age, and route of infection may all be important predictors of viral RNA shedding. Fecal swab equivalents had significantly higher virus concentrations (p<0.05) than oropharyngeal or cloacal swabs (Figure 3) and 6 mo ducks shed significantly more viral RNA (p<0.05) than 3 mo ducks for each sample type. Ducks inoculated experimentally shed viral RNA at higher rates in feces than ducks infected via contaminated water. On the other hand, experimentally inoculated ducks shed at lower rates than water transmission ducks in oropharyngeal and cloacal samples.

**Figure 4:**
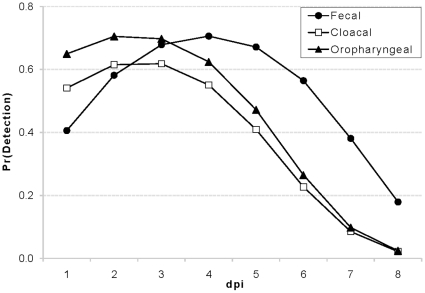
Comparison of probability of detecting H4N6 AIV RNA using 3 different sampling methods. Fecal samples show a significantly higher probability of detection 4–8 dpi. Overall, the predicted probability of detecting AIV for a single swab is 38% for a cloacal swab, 44% for an oropharyngeal swab, and 52% for a fecal swab equivalents.

While the multiple linear regression model above identified variables likely to impact shedding concentrations, the logistic model identified variables important for detecting a positive sample (e.g., during surveillance efforts). Based on minimum AIC, the best multiple logistic regression model included sample type, duck age, and dpi indicating that the probability of detecting a positive sample was associated with the type of sample, the age of the duck sampled, and the dpi the sample was collected. For 1–2 dpi, on average, fecal samples had a somewhat lower probability of detection compared to cloacal or oropharyngeal samples (Figure 4). At 3 dpi, all three sample types showed a similar probability of detecting a positive sample, but for 4–8 dpi, fecal samples showed a higher probability of detection. Overall, this model predicted that the probability of detecting AIV for a single swab collected within 8 dpi, was 33.0% (95% CI = 30.1, 47.3) for a cloacal swab, 44.2% (95% CI = 35.6, 53.1) for an oropharyngeal swab, and 52.1% (95% CI = 45.76, 58.34) for a fecal swab equivalent.

## Discussion

This is the first time that water has been documented as a source of infection for waterfowl, although it has long been hypothesized as the most likely route of AIV transmission among ducks in their natural, aquatic habitat [8], [19], [21], [28], [29], [30], [31], [32]. Additional evidence supporting this transmission route is the longevity of AIV in distilled water (estimated up to 207 days at 17°C and 102 days at 28°C) [18]. In contrast, infectious fecal material experimentally diluted in river water remained infectious to chicken embryos for only 32 days at 4°C and 7 days at 22°C [8]. Tap water used in our study was maintained at approximately 20°C and remained relatively stagnant with the only effluent and influent resulting from splashing and the daily addition of small amounts of fresh water. Under our experimental scenario, naïve ducks that were exposed to a water source used by ducks shedding AIV H4N6 also became infected (presumably through their mucous membranes encountering infectious water). Two successive passages in chicken embryos were required to detect infectious virions in the water. This suggests that the viral concentrations were too low to calculate the infectious virus titer. However, quantitative RRT-PCR estimated the viral RNA concentration in these water sources to be approximately 10^3.0^ PCR EID_50_ equivalents/mL. Evidence for aerosol transmission was not observed as two negative control ducks in a nearby pen did not become infected. The ducks presumably infected via the H4N6 contaminated water continued to shed AIV RNA at higher or similar levels as experimentally-inoculated ducks, and maintained the quantity of detectable viral RNA in the water throughout the study.

Researchers testing cloacal swabs from wild waterfowl for AIV RNA concluded that hatch-year ducks (<1 year old) were 1.7 times more likely to be positive for AIV RNA than after-hatch-year ducks (≥1 year old) [33]. A separate study experimentally inoculated different age classes of hatch-year mallards including 2 wk old, and 1-, 2-, 3- and 4- mo ducks [34]. Their findings suggested a difference in viral shedding between some of these age classes, which may impact transmission of AIV within the wild bird reservoir system. We also detected differences in viral shedding between our two age groups, 3 mo and 6 mo, with 6 mo ducks shedding significantly more viral RNA (as detected with oropharyngeal and cloacal swabs) than 3 mo ducks. This observed ‘age-effect’ between 6 mo and 3 mo ducks in our study could be due to stresses of sexual maturity and egg production or differences in receptor distribution between age groups. It was evident from egg production and gonad size at necropsy that some ducks were sexually mature, but we cannot definitively say whether or not this influenced AIV shedding. In humans, it has been reported that the distribution of influenza receptors in the lungs may change with age [35].

While cloacal swabs and oral swabs (oropharyngeal or tracheal) have historically been used to detect AIV infection in waterfowl [7], [36], our research shows that water samples and fecal samples may be useful additions or alternatives to sampling of individuals. Not only were high AIV RNA concentrations detected in feces and water samples for longer periods than cloacal or oropharyngeal swabs, but these samples require fewer resources and time for field collection. For water, our results indicate that samples exhibiting 10^2.8^ PCR EID_50_ equivalents/mL are adequate to cause infection in mallards so water collection could be used to determine the risk of AIV spread from a water body. For fecal samples, the increased probability of detecting viral RNA in feces compared with oropharyngeal or cloacal swabs indicates that collecting a fresh fecal sample when available would improve surveillance efficiency. Finally, duck age is another important variable to consider when modeling AIV transmission and/or risk. While age is often classified as “hatch-year” or “after-hatch-year,” there appear to be significant shedding differences even within hatch-year birds. While immune systems and reproductive status may play a role in AIV shedding, it is not completely understood. Therefore, more research is needed to address how AIV shedding rates (and ultimately AIV transmission) are affected by age.

We have experimentally shown that AIV can be transmitted from infected mallards to naïve mallards through a common water source, which has important ecological and surveillance implications. Instead of looking at individual ducks as the source of infection of other ducks, researchers may want to focus on water sources used by infected ducks, which have the potential to infect naïve ducks using those sources. This may be especially important during migration, where an infected water source can spread AIV across large areas by infecting ducks using those water sources as stopover areas and which then move AIV to other areas as they continue migrating.
